# Associations of five obesity indicators with cognitive performance in 30,697 Taiwan Biobank participants

**DOI:** 10.1186/s12877-022-03457-x

**Published:** 2022-11-07

**Authors:** Wan-Yu Lin

**Affiliations:** 1 Institute of Epidemiology and Preventive Medicine, College of Public Health, National Taiwan University, Taipei, Taiwan; 2 Master of Public Health Degree Program, College of Public Health, National Taiwan University, Taipei, Taiwan; 3 Department of Public Health, College of Public Health, National Taiwan University, Taipei, Taiwan

**Keywords:** Abdominal obesity, Dementia, Mini-mental state examination

## Abstract

**Background:**

Obesity adversely influences the central nervous system and cognitive functions. However, the relationship between various obesity indicators and cognitive performance remains controversial. It is unclear which obesity indicator is more relevant to cognitive impairment.

**Methods:**

The Taiwan Biobank (TWB) administered the Chinese version of the Mini-Mental State Examination (MMSE) to 30,697 participants (12,094 males and 18,603 females) aged 60 to 70 years. A total of 3,454 (11.25%) individuals with MMSE < = 24 were classified as having poor cognitive performance. This cross-sectional study investigates the associations of five obesity indicators with cognitive performance. Five separate logistic regression models were fitted for males and another five for females. Covariates adjusted in all models included age, smoking status, drinking status, regular exercise, chronic disease status (diabetes, cardiovascular diseases, heart diseases, stroke, or Parkinson’s disease), depression status, blood pressure level, total cholesterol, fasting glucose, and educational attainment. The five obesity indicators included body mass index (BMI), body fat percentage (BFP), waist circumference (WC), hip circumference (HC), and waist-hip ratio (WHR).

**Results:**

Abdominal obesity defined by WHR was significantly associated with poor cognitive performance. Male WHR > = 0.90 had a higher risk of poor cognitive performance than male WHR < 0.90 (odds ratio [OR] = 1.233; *p* = 0.007); female WHR > = 0.85 had an increased risk of poor cognitive performance compared with female WHR < 0.85 (OR = 1.221; *p* = 3.9E-4). HC and general obesity (defined by BMI and BFP) were not significantly associated with cognitive performance.

**Conclusion:**

The results consistently agreed that preventing abdominal obesity is associated with better cognitive performance in both males and females.

**Supplementary Information:**

The online version contains supplementary material available at 10.1186/s12877-022-03457-x.

## Background

The pace of population aging is increasing. According to the World Health Organization, the number of individuals over age 60 was 1 billion in 2019, estimated to be up to 1.4 billion by 2030 and 2.1 billion by 2050. For aged people, maintaining healthy cognitive function is essential for their quality of life.

Cognitive impairment is defined as difficulties in learning, remembering, concentrating, and making decisions [1]. It decreases the quality of life of aged people and is a critical risk factor for dementia [2, 3]. A tool commonly used to evaluate cognitive impairment in clinical and research settings is the Mini-Mental State Examination (MMSE) [4, 5]. MMSE was developed by Folstein et al. in 1975 [5], containing 11 items and 30 answers. Each answer is scored as one point; therefore, an MMSE test result ranges from 0 to 30. A higher MMSE score is linked to a better cognitive function. MMSE evaluates the performance of orientation, attention, calculation, memory, language, and visual-spatial skills [6].

Some studies have shown that obesity plays a critical role in the decline of cognitive function [7, 8]. Obesity and its comorbidities are risk factors for impaired cognitive performance and neurodegenerative diseases such as dementia [7, 9]. Accumulating evidence has shown that obesity adversely influences the central nervous system and cognitive functions, including learning, attention, decision making, and executive performance [10].

Despite increasing evidence of the relationship between obesity and cognitive decline, the associations of body mass index (BMI) with cognitive function are somewhat controversial [11]. Among several obesity indicators, BMI is the most commonly used indicator in previous investigations [12, 13]. It has been reported that the association between BMI and the risk of cognitive impairment is a U-shaped curve rather than a linear trend [14–16]. Some studies have shown that the overweight category is associated with the lowest risk of cognitive decline [8, 15], where ‘overweight’ indicated a BMI ranging from 23 kg/m^2^ to 27 kg/m^2^ [15] or from 24 kg/m^2^ to 28 kg/m^2^ [8]. Other studies have shown that a BMI ranging from 18.5 kg/m^2^ to 23 kg/m^2^ corresponds to the lowest cognitive impairment risk [17]. Persisting controversies remain in the association of BMI with the risk of cognitive impairment.

Moreover, some studies have also shown that a high body fat percentage (BFP) is associated with worse performance in cognitive control [18]. Abdominal obesity (indicated by a larger waist circumference [WC] or waist-hip ratio [WHR]) is associated with reduced cognitive scores [19–22] and a faster rate of cognitive decline [19, 23]. It remains unclear which obesity indicators are more relevant to cognitive functions.

Individuals with abdominal obesity (indicated by WC or WHR) present higher C-reactive protein and IL-6 concentrations [24]. Abdominal obesity, rather than general obesity, is specifically linked to these inflammatory markers [24]. Moreover, increased C-reactive protein and IL-6 are associated with dementia, identified by a meta-analysis combining seven observational studies (5,717 participants and 746 dementia cases) [25].

Given a large sample size of MMSE test results (30,697 TWB participants), I investigated the relationship between cognitive performance and five obesity indicators, including BMI, BFP, WC, hip circumference (HC), and WHR. In addition to statistical analysis, the biological plausibility of the results will be further discussed.

## Methods

### Taiwan Biobank

Since October 2012, the TWB has recruited over 100,000 community-based volunteers in Taiwan to build a biobank. Information regarding genomics and lifestyle factors was collected from each participant [26]. After signing informed consent, participants underwent physical examinations and provided urine and blood samples. Trained health professionals gathered lifestyle factors through face-to-face interviews with each participant. The obesity indicators and MMSE measurements were administered by professional nurses or medical laboratory scientists. The interviews and physical examinations for each participant were performed on the same day. The de-identified individual-level data are not publicly downloadable but are available upon application to the TWB (https://www.twbiobank.org.tw/new_web/).

By February 2021, TWB had recruited 132,720 participants aged 30 to 70 years, wherein 30,740 participants were 60 to 70 years old. A total of 30,716 of the 30,740 individuals agreed to participate in the evaluation of MMSE [27–29], including 12,108 males and 18,608 females. The body height and weight of each individual were recorded. BMI was then calculated as body weight in kilograms divided by squared body height in meters. Because body height and weight (and BMI) were basic physical examination items, I removed 14 males and 5 females without the BMI data. Through this step, 30,697 participants remained in this study, wherein 12,094 were males and 18,603 were females.

BFP is the percentage of total fat mass over total body mass. It was measured by bioelectrical impedance analysis (BIA) according to a Tanita body composition analyzer BC-420MA (Tanita Corp., Tokyo, Japan). However, 613 of the 12,094 males (5.1%) and 1,010 of the 18,603 females (5.4%) did not undergo BIA. Therefore, the inference for BFP was based on 29,074 participants, i.e., 94.7% of the 30,697 participants.

WC is the circumference of the midpoint between the iliac crest and the lowest rib (29). HC was the largest circumference around the buttocks in a standing position. Both WC and HC were measured with a nonelastic tape. WHR was then a dimensionless ratio of WC to HC. Among the 30,697 participants, only one female missed WC, HC, and WHR measurements. This female participant was still included in the analysis for BMI and BFP.

### Mini-mental state examination

The MMSE evaluates the performance of orientation, attention, calculation, memory, language, and visual-spatial skills [6]. An MMSE score of 24 or lower (adjusted by age and educational attainment [30]) is regarded as likely cognitive impairment. In contrast, a score higher than 24 generally indicates no cognitive impairment [31]. MMSE has been translated into Chinese and is widely used to diagnose dementia or cognitive impairment in many Chinese-speaking areas. It presented acceptable validity and reliability in measuring cognitive functions [27–29].

A previous study showed satisfactory agreement on MMSE scores, given a high interrater correlation coefficient of 0.998 [32]. Moreover, an MMSE score $\le$ 17 was appropriate to define suspected dementia. Based on a Chinese sample, the sensitivity and specificity according to this cutoff value (17) were 1.00 and 0.89, respectively [32]. Dementia is usually diagnosed when cognitive impairment has become severe enough to compromise occupational and social functioning [33]. However, in the TWB, only 40 males (0.3% of the 12,094 males) and 157 females (0.8% of the 18,603 females) had an MMSE score $\le$ 17.

To reach a desirable statistical power, I here investigated the associations of obesity indicators with “poor cognitive performance” (i.e., MMSE score $\le$ 24). An MMSE score ranges from 0 to 30, with a score > 24 indicating basically no cognitive impairment [31]. Therefore, I defined a score $\le$ 24 as poor cognitive performance, whereas a score > 24 was defined as normal. Here, I used the term “poor cognitive performance” instead of “cognitive impairment” because, in this stage, the MMSE score was original and had not been adjusted by age or educational attainment [30]. Age and educational attainment were adjusted in subsequent logistic regression.

### Covariates adjusted in all models

Ten covariates adjusted in all models included age, smoking status, drinking status, regular exercise, chronic disease status, depression status, blood pressure level, total cholesterol, fasting glucose, and educational attainment. These ten covariates were chosen *a priori* because they were commonly adjusted in studies of MMSE [34].

Smoking was defined as a subject who had smoked for at least 6 months and had not quit smoking when his or her phenotypes were examined. Drinking was defined as a subject having a weekly intake of more than 150cc of alcohol for at least 6 months and having not stopped drinking when his or her phenotypes were measured.

Regular exercise was defined as engaging in 30min of exercise three times a week. Exercise included leisure-time activities such as walking, brisk walking, jogging, swimming, dancing, mountain climbing, and cycling. Chronic disease status was defined as whether an individual had been diagnosed with diabetes, cardiovascular diseases, heart diseases, stroke, or Parkinson’s disease. Depression status was defined as the diagnosis of depression.

Systolic blood pressure (SBP) and diastolic blood pressure (DBP) were measured twice in a sitting position with a 5-minute rest interval. Blood pressure level was then obtained by the average of these four measurements of BP (i.e., two from SBP and two from DBP). Serum fasting glucose was obtained with a Hitachi LST008 analyzer (Hitachi High-Technologies, Tokyo, Japan) after a fast of at least 6h.

The educational attainment of each participant was surveyed through a face-to-face interview with TWB researchers. It was recorded as a number ranging from 1 to 7, with 1 indicating “illiterate”, 2 indicating “no formal education but literate”, 3 indicating “primary school graduate”, 4 indicating “junior high school graduate”, 5 indicating “senior high school graduate”, 6 indicating “college graduate”, and 7 indicating “master’s or higher degree”. All regression models coded educational attainment as an integer ranging from 1 to 7.

### Definition of obesity

This cross-sectional study assesses the associations of five obesity indicators with cognitive performance. The five obesity indicators and the MMSE score of each participant were measured on the same day (from October 2012 to February 2021). According to Taiwan’s Ministry of Health and Welfare, general obesity can be defined by BMI and/or BFP. BMI levels were categorized into four groups: underweight (BMI < 18.5 kg/m^2^), healthy weight (18.5 kg/m^2^ < = BMI < 24 kg/m^2^), overweight (24 kg/m^2^ < = BMI < 27 kg/m^2^), and obese (BMI > = 27 kg/m^2^). Concerning the criterion of BFP, males with BFP > = 25% or females with BFP > = 30% were classified into the obesity group.

According to Taiwan’s Ministry of Health and Welfare, abdominal obesity can be defined either way: (1) WC $\ge$ 90 cm for males or WC $\ge$ 80 cm for females; (2) WHR $\ge$ 0.90 for males or WHR $\ge$ 0.85 for females. Here, I assessed the associations of both definitions of abdominal obesity with poor cognitive performance.

### Statistical analysis

Males and females are different in body composition and risks of cognitive impairment [35]. Females generally have a larger BFP than males, as this is the case in the current study (Table 1). Moreover, females showed a significant higher prevalence of cognitive impairment than males after age 75 (62.7% vs. 45.4%, *P*-value < 0.005) [35]. To explore sex-specific obesity indicators associated with cognitive performance, I analyzed male and female individuals with separate logistic regression models. With the logistic regression model, the status of cognitive performance (poor if MMSE $\le$ 24 vs. normal if MMSE > 24) was regressed on BMI/BFP/WC/WHR categories in separate logistic regression models (Because HC has not been used to define obesity, it was not investigated in this step). In all models, I adjusted for the ten covariates mentioned above, including age, smoking status, drinking status, regular exercise, chronic disease status, depression status, blood pressure level, total cholesterol, fasting glucose, and educational attainment.

**Table 1:** **Basic characteristics of the 30,697 TWB participants**

	Males	Females	***P***-value ^1^
Total	12,094 (39.4%)	18,603 (60.6%)	
Age (years)	64.2$\pm$2.9	63.8$\pm$2.8	< 0.001
BMI (kg/m^2^)	24.9$\pm$3.1	24.0$\pm$3.5	< 0.001
Underweight (yes or no) BMI < 18.5 kg/m^2^	169 (1.4%)	563 (3.0%)	< 0.001
Healthy weight (yes or no) 18.5 < = BMI < 24 kg/m^2^	4,632 (38.3%)	9,788 (52.6%)	< 0.001
Overweight (yes or no) 24 < = BMI < 27 kg/m^2^	4,622 (38.2%)	5,027 (27.0%)	< 0.001
Obesity (yes or no) BMI > = 27 kg/m^2^	2,671 (22.1%)	3,225 (17.4%)	< 0.001
Body fat percentage (%)	22.4$\pm$5.1	32.9$\pm$6.1	< 0.001
Obesity defined by BFP ($\ge$25% for males; $\ge$30% for females)	3,382 (29.5% among 11,481 males with BFP)	12,238 (69.6% among 17,593 females with BFP)	< 0.001
Waist circumference (cm)	88.0$\pm$8.5	83.3$\pm$9.6	< 0.001
Abdominal obesity defined by WC ($\ge$90 cm for males; $\ge$80 cm for females)	4,899 (40.5%)	11,623 (62.5%)	< 0.001
Hip circumference (cm)	95.8$\pm$5.8	94.5$\pm$6.8	< 0.001
Waist-hip ratio	0.92$\pm$0.05	0.88$\pm$0.07	< 0.001
Abdominal obesity defined by WHR ($\ge$0.90 for males; $\ge$0.85 for females)	7,630 (63.1%)	12,319 (66.2%)	< 0.001
Drinking (yes or no) ^2^	1,408 (11.6%)	236 (1.3%)	< 0.001
Smoking (yes or no) ^3^	1,624 (13.4%)	192 (1.0%)	< 0.001
Regular exercise (yes or no) ^4^	7,767 (64.2%)	11,564 (62.2%)	< 0.001
Status of chronic diseases ^5^	3,990 (33.0%)	4,889 (26.3%)	< 0.001
Status of depression	327 (2.7%)	861 (4.6%)	< 0.001
MMSE score	27.6$\pm$2.3	27.3$\pm$2.6	< 0.001
Poor cognitive performance (MMSE score < = 24)	1,054 (8.7%)	2,400 (12.9%)	< 0.001
Education			
Refuse to answer this question	2 (0.02%)	8 (0.04%)	0.351
Illiterate	8 (0.07%)	168 (0.90%)	< 0.001
No formal education but literate	7 (0.06%)	41 (0.22%)	< 0.001
Primary school graduate	1,106 (9.14%)	3,456 (18.58%)	< 0.001
Junior high school graduate	1,053 (8.71%)	2,623 (14.10%)	< 0.001
Senior high school graduate	3,202 (26.48%)	6,156 (33.09%)	< 0.001
College graduate	5,570 (46.05%)	5,466 (29.39%)	< 0.001
Master’s or higher degree	1,146 (9.47%)	685 (3.68%)	< 0.001

Data are presented in *n* (%) or mean$\pm$SD.

^1^*P*-value of testing the mean difference between males and females, based on the proportion test (for BMI categories: underweight, healthy weight, overweight, and obese; drinking; smoking; regular exercise; status of chronic diseases; status of depression; MMSE score < = 24) or the two-sample *t*-test (for other variables). For *P*-values less than 0.001, I reported it as “< 0.001”

^2^ Drinking was defined as a person having a weekly intake of more than 150 mL of alcohol for at least 6 months and having not stopped drinking at the time he or she participated in the TWB.

^3^ Smoking was defined as a person who had smoked cigarettes for at least 6 months and had not quit smoking at the time he or she participated in the TWB.

^4^ Regular exercise was defined as performing 30min of exercise three times a week. Exercise included leisure-time activities such as swimming, jogging, cycling, mountain climbing, dancing, and weight training

^5^ Status of chronic diseases was defined as whether an individual had diabetes, cardiovascular diseases, heart diseases, stroke, or Parkinson’s disease

As a sensitivity analysis, obesity indicators were also treated as continuous scales in the regression models. The cognitive performance status was regressed on the *z*-score transformation of each obesity indicator separately while adjusting for the same ten covariates. Through the *z*-score transformation, the odds ratios (ORs) were independent of the units of various obesity indicators (kg/m^2^ for BMI; % for BFP; cm for WC and HC). Although HC has not been used to define obesity, it is the denominator of WHR. Therefore, it was also investigated in this step. The effect sizes of various obesity indicators can be compared directly. Because the obesity indicators were investigated in separate regression models, I adjusted multiple testing with the false discovery rate (FDR) control. *P*-values with Benjamini-Hochberg FDR [36] < 5% were considered statistically significant.

Some studies defined cognitive performance as poor if MMSE $\le$ 25 vs. normal if MMSE > 25 [37, 38]. As another sensitivity analysis, I performed all the abovementioned analyses according to this cutoff ($\le$ 25 vs. > 25). All analyses were performed using R statistical software (version 4.1.1; The R Project for Statistical Computing; http://www.r-project.org/).

## Results

### Basic characteristics

Table 1 summarizes the characteristics of 12,094 males (39.4%) and 18,603 females (60.6%). Except for BFP, male participants, on average, had larger values of the obesity indicators than did female participants. The percentages of alcohol consumption, cigarette smoking, and regular exercise were higher in males than females. Males, on average, had higher educational attainment than females. A total of 55.5% of males and 33.1% of females had college or higher degrees. Regarding the outcome measure, fewer males were categorized as having poor cognitive performance (MMSE score $\le$ 24) than females (8.7% vs. 12.9%, Table 1).

### BMI/BFP/WC/WHR categories and cognitive performance

Most male participants had healthy weights (38.3%, 18.5 kg/m^2^ < = BMI < 24 kg/m^2^) or were overweight (38.2%, 24 kg/m^2^ < = BMI < 27 kg/m^2^), whereas only 1.4% of males were underweight (BMI < 18.5 kg/m^2^). In contrast, more female participants had healthy weights (52.6%, Table 1).

By treating the healthy weight category as the reference group, none of the four BMI categories presented significantly different risks of poor cognitive performance (Table 2). The underweight group showed the lowest risk (odds ratio [OR] < 1.0), whereas the obesity group (BMI > = 27 kg/m^2^) presented the highest risk, although these ORs were not significantly different from 1.0. Generally, the risks of poor cognitive performance increased with BMI levels, and this result was consistently observed in males and females (Table 2).

**Table 2:** **Odds ratio of poor cognitive performance for various BMI (or BFP, WC, WHR) categories compared to the healthy BMI (or BFP, WC, WHR) group (** ***P*** **-values with false discovery rates < 5% are shown in bold)**

Definition of obesity	Male participants			Female participants		
	Odds ratio ^1^	95% C.I.	***P***-value	Odds ratio^1^	95% C.I.	*P*-value
General obesity defined by BMI ^2^						
Underweight (yes vs. no) BMI < 18.5 kg/m^2^	0.822	[0.406, 1.508]	0.554	0.814	[0.563, 1.146]	0.254
Overweight (yes vs. no) 24 kg/m^2^ < = BMI < 27 kg/m^2^	1.128	[0.963, 1.322]	0.135	1.031	[0.920, 1.155]	0.600
Obesity (yes vs. no) BMI > = 27 kg/m^2^	1.158	[0.966, 1.385]	0.111	1.099	[0.966, 1.250]	0.150
General obesity defined by BFP (yes vs. no) ^3^ BFP $\ge$ 25% for males BFP $\ge$ 30% for females	1.111	[0.955, 1.290]	0.171	1.035	[0.920, 1.165]	0.569
Abdominal obesity defined by WC (yes vs. no) ^4^ WC $\ge$ 90 cm for males WC $\ge$ 80 cm for females	1.168	[1.018, 1.340]	0.027	1.214	[1.091, 1.351]	**3.9E-4**
Abdominal obesity defined by WHR (yes vs. no) ^5^ WHR $\ge$ 0.90 for males WHR $\ge$ 0.85 for females	1.233	[1.061, 1.436]	**0.007**	1.221	[1.094, 1.364]	**3.9E-4**

^1^ In all logistic regression models, I adjusted for ten covariates: age, smoking status (yes vs. no), drinking status (yes vs. no), regular exercise (yes vs. no), chronic disease status (yes vs. no), depression status (yes vs. no), blood pressure level, total cholesterol, fasting glucose, and educational attainment (1, 2, …, or 7)

^2^ Reference group: the healthy weight group (18.5 kg/m^2^ < = BMI < 24 kg/m^2^).

^3^ Reference group: BFP < 25% for males; BFP < 30% for females

^4^ Reference group: WC < 90 cm for males; WC < 80 cm for females

^5^ Reference group: WHR < 0.90 for males; WHR < 0.85 for females

The mean BFP in females was 32.9$\pm$6.1 (%), and 69.6% of female participants were categorized as obese according to the BFP criterion (Table 1). Fortunately, although a large proportion of female participants had a BFP > = 30%, female BFP > = 30% was not a significant risk factor for poor cognitive performance (OR = 1.035, *p* = 0.569, Table 2).

Abdominal obesity can be defined by WC and/or WHR. Different from general obesity (indicated by BMI or BFP), abdominal obesity was significantly associated with poor cognitive performance, especially that defined by WHR. The ORs were 1.233 (95% CI = 1.061 ~ 1.436, *p* = 0.007) and 1.221 (95% CI = 1.094 ~ 1.364, *p* = 3.9E-4) for male WHR > = 0.90 and female WHR > = 0.85, respectively (Table 2). Moreover, the OR was 1.214 (95% CI = 1.091 ~ 1.351, *p* = 3.9E-4) for female WC > = 80 cm (Table 2).

### Continuous BMI/BFP/WC/HC/WHR values and cognitive performance

In addition to assessing the associations of cognitive performance with various BMI/BFP/WC/WHR categories, I also treated the five obesity indicators as continuous scales in the association analysis. The *z*-score transformation was performed on each obesity measure before fitting the logistic regression. Table 3 shows the ORs of poor cognitive performance by increasing one standard deviation (SD) of each obesity indicator. Again, WHR provided the most significant association with cognitive performance. For males, an increase of one SD in WHR (0.05) was significantly associated with an increase in the risk of poor cognitive performance (OR = 1.107, 95% CI = 1.033 ~ 1.187, *p* = 4.0E-3). For females, every SD increase in WHR (0.07) was significantly associated with an increase in the risk of poor cognitive performance (OR = 1.136, 95% CI = 1.081 ~ 1.194, *p* = 5.9E-7), and every SD increase in WC (9.6 cm) was also significantly associated with an increase in the risk of poor cognitive performance (OR = 1.109, 95% CI = 1.055 ~ 1.165, *p* = 4.3E-5, Table 3).

**Table 3:** **Odds ratio of poor cognitive performance by increasing one SD of each obesity indicator (** ***P*** **-values with false discovery rates < 5% are shown in bold)**

	Male participants			Female participants		
	Odds ratio	95% C.I.	***P***-value	Odds ratio	95% C.I.	***P***-value
BMI ^1^	1.068	[0.998, 1.142]	0.057	1.059	[1.008, 1.112]	0.023
Body fat percentage ^1^	1.078	[1.003, 1.158]	0.042	1.053	[0.999, 1.110]	0.055
Waist circumference ^1^	1.067	[0.997, 1.142]	0.061	1.109	[1.055, 1.165]	**4.3E-5**
Hip circumference ^1^	1.007	[0.941, 1.076]	0.849	1.028	[0.979, 1.078]	0.266
Waist-hip ratio ^1^	1.107	[1.033, 1.187]	**4.0E-3**	1.136	[1.081, 1.194]	**5.9E-7**

^1^ The *z*-score transformation was performed on each obesity measure before fitting the logistic regression. In all logistic regression models, I adjusted for ten covariates: age, smoking status (yes vs. no), drinking status (yes vs. no), regular exercise (yes vs. no), chronic disease status (yes vs. no), depression status (yes vs. no), blood pressure level, total cholesterol, fasting glucose, and educational attainment (1, 2, …, or 7)

Some studies used another MMSE cutoff to define cognitive performance, i.e., poor if MMSE $\le$ 25 vs. normal if MMSE > 25 [37, 38]. The results corresponding to this cutoff also agreed that abdominal obesity is more relevant to cognitive performance than general obesity (Supplementary Materials, Tables S1-S2).

## Discussion

This study showed that WHR was most relevant to cognitive performance among five commonly used obesity indicators. The results consistently agreed that preventing abdominal obesity is associated with better cognitive performance in both sexes. WC is a straightforward measurement for abdominal obesity. However, WC alone may underestimate the risk of abdominal obesity for smaller people. In contrast, WHR adjusts WC with HC, becoming a better health indicator than WC in some situations [39–41]. For example, based on 1,189 adults aged 70–79 years at baseline, 12-year all-cause mortality was associated with WHR rather than WC or BMI [39].

This study has two significant strengths: the availability of various obesity indicators and a much larger sample size for MMSE results in a single study (30,697 individuals) compared with previous studies [8, 42]. Cognitive decline can start from age 45 [43]. Although these 30,697 individuals (aged 60 to 70 years) were relatively young considering cognitive impairment, identifying the obesity indicator most associated with cognitive performance at age 60–70 can help prevent cognitive impairment later.

Obesity indicators were treated as continuous scales (Table 3) and several categories (Table 2) according to Taiwan’s Ministry of Health and Welfare recommendations. Results consistently agreed that, among the five obesity indicators, WHR was most relevant to cognitive performance. WC was generally the second indicator that was associated with cognitive performance. These results highlighted the importance of abdominal obesity to the risk of cognitive impairment.

Both the results from males and females showed the tendency of increased risk of poor cognitive performance given elevated BMI levels, but these associations were not statistically significant (Table 2). Due to this nonsignificant result, the associations of obesity with cognitive function may be overlooked. This study illustrates that abdominal obesity, rather than general obesity, is associated with cognitive performance.

According to the criteria of Taiwan’s Ministry of Health and Welfare, many female participants were in the general obesity category according to BFP (69.6%, Table 1) and abdominal obesity by WHR (66.2%). However, only abdominal obesity by WHR was significantly associated with an increased risk of poor cognitive performance (OR = 1.221, 95% CI = 1.094 ~ 1.364, *p* = 3.9E-4, Table 2). In contrast, general obesity by BFP was found to be of little relevance to poor cognitive performance (OR = 1.035, 95% CI = 0.920 ~ 1.165, *p* = 0.569). A large WHR is a threat to cognitive health.

A previous study used the same criterion for WHR (male WHR $\ge$ 0.90; female WHR $\ge$ 0.85) to define abdominal obesity, and the investigators also found a significant association between abdominal obesity and cognitive impairment (OR = 1.532, 95% CI = 1.037~2.263, *p* = 0.032) [8]. This result was in line with the results shown here. However, the current work has three strengths over the previous study [8]. First, this work was based on a much larger sample size (30,697) than that of the previous research (1,100) [8]. Second, more obesity indicators were assessed (five) than in the previous research (only BMI and WHR) [8]. Finally, I performed an analysis within each sex stratum. A recent study has shown that aging is associated with different obesity indicators in males and females [44]. I performed a sex-specific analysis to clarify whether this is also the case in cognitive performance.

Abdominal obesity indicates excess truncal (particularly visceral) fat [45], which specifically increases the risk of developing insulin resistance [46]. Insulin resistance further drives metabolic syndromes and cognitive declines [47]. By analyzing the MMSE results of more than 30,000 TWB individuals, this study confirmed the link between abdominal obesity and poor cognitive performance. Abdominal obesity is a risk factor for poor cognitive performance independent of age, educational attainment, smoking, drinking, regular exercise, depression, blood pressure level, total cholesterol, fasting glucose, and chronic diseases (diabetes, cardiovascular diseases, heart diseases, stroke, or Parkinson’s disease).

Finally, the main limitation of this work is that it is a cross-sectional study, and the associations observed here cannot be explained as causality. Residual confounding and reverse causation are possible. Furthermore, the lack of association of BMI (or BFP) with poor cognitive performance could be because of insufficient power. Medication information was not collected by the TWB, and therefore it was not adjusted in the analyses. An even more extensive study will be needed to replicate these results.

## Electronic supplementary material

Below is the link to the electronic supplementary material.

Supplementary material: Table S1. Odds ratio of “poor cognitive performance” (MMSE ≤ 25) for various BMI (or BFP, WC, WHR) categories compared to the healthy BMI (or BFP, WC, WHR) group (P-values with false discovery rates < 5% were shown in bold)
Table S2. Odds ratio of “poor cognitive performance” (MMSE ≤ 25) by increasing one SD of each obesity indicator (P-values with false discovery rates < 5% were shown in bold)

## Data Availability

The individual-level TWB data supporting this study’s findings are available upon application to TWB (https://www.twbiobank.org.tw/new_web/). TWB approved my application to access the data on February 18, 2020 (application number: TWBR10810-07; principal investigator: Wan-Yu Lin).

## Abbreviations

BFP: body fat percentage.
BMI: body mass index.
FDR: false discovery rate.
MMSE: Mini-Mental State Examination.
HC: hip circumference.
TWB: Taiwan Biobank.
WC: waist circumference.
WHR: waist-hip ratio.
