# Supplementary material for: Associations of five obesity indicators with cognitive performance in 30,697 Taiwan Biobank participants
Source: BMC Geriatr. 2022 Nov 7;22:839. doi: 10.1186/s12877-022-03457-x (PMC9641815; doi:10.1186/s12877-022-03457-x)
Supplement: Supplementary file 1 — Supplementary material: Table S1. Odds ratio of “poor cognitive performance” (MMSE ≤ 25) for various BMI (or BFP, WC, WHR) categories compared to the healthy BMI (or BFP, WC, WHR) group (P-values with false discovery rates < 5% were shown in bold) Table S2. Odds ratio of “poor cognitive performance” (MMSE ≤ 25) by increasing one SD of each obesity indicator (P-values with false discovery rates < 5% were shown in bold) [file 12877_2022_3457_MOESM1_ESM.pdf]

Supplementary Materials of

Associations of five obesity indicators with cognitive performance in 30,697 Taiwan Biobank participants

Wan-Yu Lin [1,2,3]*

[1] Institute of Epidemiology and Preventive Medicine, College of Public Health, National Taiwan University, Taipei, Taiwan

[2] Master of Public Health Degree Program, College of Public Health, National Taiwan University, Taipei, Taiwan

[3] Department of Public Health, College of Public Health, National Taiwan University, Taipei, Taiwan

Running title: Obesity and cognitive performance

* Corresponding author: Wan-Yu Lin, Ph.D.

Wan-Yu Lin, Ph.D. (http://orcid.org/0000-0002-3385-4702)

Room 501, No. 17, Xu-Zhou Road, Taipei 100, Taiwan

Phone/Fax: +886-2-33668106; E-mail: linwy@ntu.edu.tw

Table S1. Odds ratio of "poor cognitive performance" (MMSE $\leq$ 25) for various BMI (or BFP, WC, WHR) categories compared to the healthy BMI (or BFP, WC, WHR) group (*P*-values with false discovery rates < 5% were shown in bold)

Table S2. Odds ratio of "poor cognitive performance" (MMSE $\leq$ 25) by increasing one SD of each obesity indicator (*P*-values with false discovery rates < 5% were shown in bold)

**Obesity definition**	Male participants			Female participants		
	Odds ratio [1]	95% C.I.	*P*-value	Odds ratio [1]	95% C.I.	*P*-value
**General obesity defined by BMI [2]**						
**Underweight (yes vs. no)** **BMI < 18.5 kg/m^2**	0.800	[0.473, 1.288]	0.381	0.945	[0.714, 1.234]	0.684
**Overweight (yes vs. no)** **24 kg/m^2 <= BMI < 27 kg/m^2**	1.045	[0.923, 1.183]	0.488	1.020	[0.926, 1.124]	0.683
**Obesity (yes vs. no)** **BMI >= 27 kg/m^2**	1.101	[0.954, 1.270]	0.186	1.105	[0.988, 1.234]	0.079
**General obesity defined by BFP (yes vs. no) [3]** **BFP ≥ 25% for males** **BFP ≥ 30% for females**	1.116	[0.989, 1.257]	0.073	1.011	[0.917, 1.116]	0.828
**Abdominal obesity defined by WC (yes vs. no) [4]** **WC ≥ 90 cm for males** **WC ≥ 80 cm for females**	1.109	[0.994, 1.237]	0.065	1.166	[1.066, 1.275]	**7.6E-4**
**Abdominal obesity defined by WHR (yes vs. no) [5]** **WHR ≥ 0.90 for males** **WHR ≥ 0.85 for females**	1.151	[1.024, 1.294]	0.019	1.179	[1.076, 1.293]	**4.4E-4**

Table S1. **Odds ratio of “poor cognitive performance” (MMSE ≤ 25) for various BMI (or BFP, WC,**

WHR) categories compared to the healthy BMI (or BFP, WC, WHR) group (*P*-values with false discovery rates < 5% were shown in bold)

[1] In all logistic regression models, I adjusted for ten covariates: age, smoking status (yes vs. no), drinking status (yes vs. no), regular exercise (yes vs. no), chronic disease status (yes vs. no), depression status (yes vs. no), blood pressure level, total cholesterol, fasting glucose, and educational attainment (1, 2, …, or 7).

[2] Reference group: the healthy weight group (18.5 kg/m^2 <= BMI < 24 kg/m^2).

[3] Reference group: BFP < 25% for males; BFP < 30% for females.

[4] Reference group: WC < 90 cm for males; WC < 80 cm for females.

[5] Reference group: WHR < 0.90 for males; WHR < 0.85 for females.

	Male participants			Female participants		
	Odds ratio	95% C.I.	*P*-value	Odds ratio	95% C.I.	*P*-value
**BMI** [1]	1.044	[0.989, 1.101]	0.121	1.056	[1.012, 1.101]	**0.012**
**Body fat percentage** [1]	1.053	[0.995, 1.115]	0.076	1.045	[0.999, 1.093]	0.053
**Waist circumference** [1]	1.038	[0.983, 1.096]	0.180	1.096	[1.050, 1.143]	**2.4E-5**
**Hip circumference** [1]	0.989	[0.937, 1.043]	0.678	1.038	[0.996, 1.082]	0.073
**Waist-hip ratio** [1]	1.076	[1.018, 1.137]	**0.009**	1.102	[1.056, 1.150]	**7.3E-6**

Table S2. **Odds ratio of "poor cognitive performance" (MMSE $\leq$ 25) by increasing one SD of each obesity indicator (*P*-values with false discovery rates < 5% were shown in bold)**

[1] The *z*-score transformation was performed on each obesity measure before fitting the logistic regression. In all logistic regression models, I adjusted for ten covariates: age, smoking status (yes vs. no), drinking status (yes vs. no), regular exercise (yes vs. no), chronic disease status (yes vs. no), depression status (yes vs. no), blood pressure level, total cholesterol, fasting glucose, and educational attainment (1, 2, …, or 7).
